# Sex differences in Parkinson’s disease-related non motor symptoms: a focus on sleep problems

**DOI:** 10.1007/s13760-024-02535-8

**Published:** 2024-04-04

**Authors:** Roberta Bovenzi, Matteo Conti, Valentino De Franco, Mariangela Pierantozzi, Tommaso Schirinzi, Rocco Cerroni, Alessandro Stefani, Nicola Biagio Mercuri, Claudio Liguori

**Affiliations:** 1 Department of Systems Medicine, University of Rome “Tor Vergata”, Via Montpellier 1, 00133 Rome, Italy; 2 Department of Systems Medicine, Parkinson’s Disease Unit, University Hospital of Rome “Tor Vergata”, Via Montpellier 1, 00133 Rome, Italy; 3 Sleep Medicine Centre, University Hospital of Rome “Tor Vergata”, Via Montpellier 1, 00133 Rome, Italy

**Keywords:** Parkinson’s disease, Sex, Gender, Sleep problems, Non motor symptoms

## Abstract

Parkinson’s disease (PD) symptomatology differs between females and males, yet the contribution of sex on sleep problems needs further analysis. Here, we aimed to investigate sex-specific patterns in the relationship between sleep problems, assessed using the Parkinson’s disease sleep scale (PDSS-2), non motor symptoms (NMS), measured by the NMS scale (NMSS), and health-related quality of life (HR-QoL), evaluated by the Parkinson’s disease questionnaire (PDQ-39), in a large cohort of PD patients. One-hundred-fifty-four PD patients were included in the study. Female PD patients (n = 62) exhibited a higher prevalence of sleep problems than males (n = 92), with nocturnal motor-related sleep issues being the most frequent. Sleep disturbances differently correlated with a range of NMS between the two sexes. In females, sleep problems mostly correlated with pain; on the other hand, sleep disturbances were linked to a frailer phenotype characterized by global dysautonomia, perception disturbances, and impaired cognitive function in males. Whether female PD patients experienced a lower HR-QoL than males, sleep disturbances were associated with a worse HR-QoL in both sexes. In conclusion, sleep problems in PD differently burden the two sexes, suggesting possible different etiopathogenesis, diagnostic investigations, and possibly tailored approaches.

**Supplementary Information:**

The online version contains supplementary material available at 10.1007/s13760-024-02535-8.

## Introduction

Parkinson’s disease (PD) is a disabling neurodegenerative disorder characterized by primary loss of dopaminergic nigral cells and widespread accumulation of α-synuclein-containing Lewy Bodies (LBs) [1].

In addition to the cardinal motor symptoms, PD is burdened by a wide range of non-motor symptoms (NMS), often preceding the motor ones, and significantly affecting the overall health-related quality of life (HR-QoL), in particular when motor fluctuations occur [2]. Among them, sleep disturbances are estimated to occur in up to 90% of PD patients, encompassing REM sleep behavior disorder (RBD), insomnia, restless leg syndrome (RLS), nocturia, sleep-disordered breathing, and excessive daytime sleepiness (EDS) [3].

Epidemiologic, clinical features, and pathophysiological changes significantly differ between females and males with PD, suggesting sex is a critical determinant in disease development [4, 5]. PD is almost twice as common in males, who also display greater motor impairment, postural deformities, and cognitive decline, suggesting a peculiar vulnerability to the disease [4]. On the contrary, women with PD appear to have slower motor progression, possibly driven by the beneficial effect of female sex hormones on the degenerating nigral cells, though greater rates of mood disturbances, pain, and overall worse HR-QoL, implying that other factors beyond biology play a fundamental role in the sex dimorphism of the disease [6, 7]. Despite an increasing interest in the role of sex in PD, a “gender gap” remains an unmet need in PD research and routine clinical practice. Similarly, there is limited investigation into sex-specific differences in sleep patterns.

The Parkinson’s disease sleep scale- second version (PDSS-2) is a reliable and comprehensive tool designed to screen and quantify sleep complaints among individuals with PD [8]. Its transformation from a visual analogue scale to a frequency-based measure has significantly improved its usability, making it more accessible for patients and researchers conducting clinical studies. Despite these advancements, there remains a paucity of research studies utilizing the PDSS-2 to investigate sleep problems in PD.

This observational study aimed to delve into sex-specific differences in sleep-related problems, as assessed by the PDSS-2 scale, their connections with other NMS, and the HR-QoL within a large cohort of PD patients.

## Methods

### Study population

This single-Centre, observational study involved 154 PD outpatients consecutively assessed at the PD Unit of Tor Vergata University Hospital (Rome—Italy) from January 2019 to July 2019. The main inclusion criterion was the diagnosis of idiopathic PD made by a movement disorder specialist according to the 2015 MDS criteria [9]. Exclusion criteria were: atypical/secondary parkinsonism; PD-dementia with a Mini Mental State Examination <24; other neurological and medical diseases affecting sleep; shift work or other conditions interfering with a regular sleep–wake cycle; unavailability to fill the self-administered clinical scales.

This study was conducted following the principles of the Helsinki Declaration. The local ethics committee approved the study.

### Assessments

Socio-demographic and clinical history data were collected from all patients. PD motor severity was assessed through the MDS Unified Parkinson’s Disease Rating Scale part 3 (MDS-UPDRS-III) [10] and the Hoehn and Yahr scale (H&Y) [11]. Clinical evaluation was performed in “ON state”, under the effect of habitual antiparkinsonian therapy. The personal levodopa equivalent daily dose (LEDD, mg/day) was calculated in each patient using conventional formula [12].

Sleep problems were investigated through the PDSS-2 scale, which consists of 15 questions investigating various sleep and nocturnal disturbances combined in five domains: nocturnal movement-related problems (PDSS-2 I), quality of sleep (PDSS-2 II), dreaming distress (PDSS-2 III), fragmentation of sleep (PDSS-2 IV), and insomnia symptoms (PDSS-2 V) [8]. A cut-off of 18 has been identified in a European PD population as a score at which nocturnal problems are considered clinically significant and require further diagnostic workup and treatment [13].

The NMS scale (NMSS) was used for non-motor disturbances. NMSS is a 30-item scale including the following nine domains: cardiovascular, sleep/fatigue, mood/cognition, perceptual problems, attention/memory, gastrointestinal, urinary, sexual function, and miscellaneous (pain, smell, weight, and sweating) [14]. Each item of the “miscellaneous” domain was considered individually.

The Parkinson’s Disease Questionnaire-39 (PDQ-39), a self-administered questionnaire, was used to assess the impact of PD on HR-QoL; it contains 39 items covering eight domains: mobility, activities of daily living (ADL), emotional well-being, stigma, social support, cognition, communication, and bodily discomfort [15].

### Statistical analysis

Descriptive statistics were used to describe the demographic and clinical characteristics of the sample. A one-way-ANCOVA adjusted for age, disease duration, and LEDD was used to compare clinical scales between the two sexes. The correlations between PDSS-2, PDQ-39, and NMSS scores were performed using simple Pearson correlation. A cut-off score of 18 at the PDSS-2 was used to define clinically relevant PD-specific sleep disturbances. Female and male PD groups were divided into two subgroups to differentiate poor sleepers (PDSS-2 total score ≥18) from good sleepers (PDSS-2 total score <18). A chi-square test was used to compare this qualitative variable (poor sleepers or not) in the two sexes. A one-way-ANCOVA adjusted for age, disease duration, and LEDD was used to compare quantitative variables between the two subgroups found in each sex and between female poor sleepers and male poor sleepers. A p-value <0.05 was considered statistically significant. Statistical analysis was performed using IBM SPSS statistics 26.

## Results

This observational study included 154 patients with PD, 92 males (59.7%) and 62 females (40.3%), with a mean age of 68.8 ± 9.2 years and disease duration of 6.0 ± 5.2 years. The study population’s demographic, clinical, and biochemical data are summarized in Table 1.

**Table 1:** The table shows the main demographic and clinical data of females and males with PD

	Females (n = 62)	Males (n = 92)	p-Value
Age	68.7 ± 10.1	68.8 ± 8.7	NS
AAO	63.5 ± 10.9	62.3 ± 10.0	NS
Disease duration	5.2 ± 4.9	6.5 ± 5.3	NS
TD/RA/M	7/28/22	17/43/25	NS
LEDD	322.3 ± 322.0	457.5 ± 393.7	NS
H&Y stage	2.2 ± 0.8	2.3 ± 0.8	NS
MDS-UPDRS III	24.4 ± 13.4	25.0 ± 11.6	NS
PDSS-2 motor-related issues	5.0 ± 4.6	3.3 ± 3.7	*p = 0.025
PDSS-2 quality of sleep	2.9 ± 2.2	2.6 ± 2.3	NS
PDSS-2 dreaming distress	1.5 ± 2.1	1.7 ± 2.4	NS
PDSS-2 sleep fragmentation	6.9 ± 3.7	6.7 ± 3.1	NS
PDSS-2 insomnia	2.0 ± 2.3	1.6 ± 2.1	NS
PDSS-2 total	18.2 ± 10.7	15.9 ± 8.9	*p = 0.002
NMSS cardiovascular	1.0 ± 0.4	1.9 ± 0.3	NS
NMSS sleep/fatigue	11.4 ± 10.9	10.8 ± 9.2	NS
NMSS mood/cognition	11.3 ± 7.5	10.3 ± 1.4	NS
NMSS perceptual problems	0.7 ± 03	0.6 ± 0.2	NS
NMSS attention/memory	4.1 ± 0.9	3.7 ± 0.8	NS
NMSS gastrointestinal	3.7 ± 0.6	3.8 ± 0.5	NS
NMSS urinary	11.1 ± 1.3	10.4 ± 1.1	NS
NMSS sexual function	4.0 ± 0.7	3.5 ± 0.6	NS
NMSS pain	2.3 ± 3.5	1.1 ± 2.0	*p = 0.007
NMSS smell	4.4 ± 0.4	3.5 ± 0.4	NS
NMSS weight	0.3 ± 0.1	0.1 ± 0.1	NS
NMSS sweating	1.3 ± 0.3	0.4 ± 0.2	*p = 0.001
NMSS total	56.9 ± 41.4	53.4 ± 40.0	NS
PDQ-39 mobility	13.8 ± 11.2	12.0 ± 11.6	NS
PDQ-39 ADL	6.1 ± 6.1	6.2 ± 5.8	NS
PDQ-39 emotional well-being	10.0 ± 5.6	7.3 ± 4.3	*p = 0.002
PDQ-39 stigma	3.1 ± 3.6	2.4 ± 3.0	NS
PDQ-39 social	0.7 ± 1.5	0.5 ± 1.5	NS
PDQ-39 cognition	3.5 ± 3.4	3.1 ± 2.9	NS
PDQ-39 communication	2.0 ± 2.4	1.6 ± 2.2	NS
PDQ-39 bodily discomfort	4.8 ± 2.9	3.4 ± 2.7	*p = 0.001
PDQ-39 total	43.0 ± 26.9	35.9 ± 24.2	*p = 0.029

n, number; AAO, age at onset; MDS-UPDRS, Movement Disorder Society Unified Parkinson’s Disease Rating Scale; TD, tremor dominant; RA, rigid-akinetic; M, mixed; PDSS-2, Parkinson’s Disease Sleep Scale-2; NMSS, Non Motor Symptoms Scale; PDQ-39; Parkinson’s disease Questionnaire. Age, age at onset, and disease duration are expressed in years. *Significant differences between groups. Comparison of demographic and clinical scales between sexes was performed using a one-way-ANCOVA adjusted for age, disease duration, and LEDD for quantitative variables and Chi-square test statistics for qualitative variables

### Comparison of main clinical features between females and males with PD

Males and females did not differ in main demographic and clinical characteristics, including age, age at onset, and disease duration. No differences were found in motor features as assessed through the H&Y and the MDS-UPDRS part III scales and the levodopa requirements (LEDD values).

PDSS-2 values were available for n = 87 males and n = 60 females. Female patients had higher PDSS-2 total [F = 5.16, p = 0.025] and PDSS-2 nocturnal movement-related problems [F = 9.56, p = 0.002] than males. Distributing PD patients according to the PDSS-2 cut-off, we found that 29 females (48.3%) and 28 males (32.2%) were considered poor sleepers (χ^2^ = 3.901, p = 0.048).

NMSS values were available for n = 83 males and n = 57 females. Female PD patients had higher NMS pain [F = 7.38, p = 0.007] and sweating sub-scores [F = 6.74, p = 0.001] compared to males.

Finally, PDQ39 values were available for n = 83 males and n = 57 females. Female PD patients had higher PDQ-39 total scores [F = 4.88, p = 0.029], emotional well-being [F = 9.53, p = 0.002], and bodily discomfort sub-scores [F = 12.59, p = 0.001] compared to males.

**Fig. 1:**
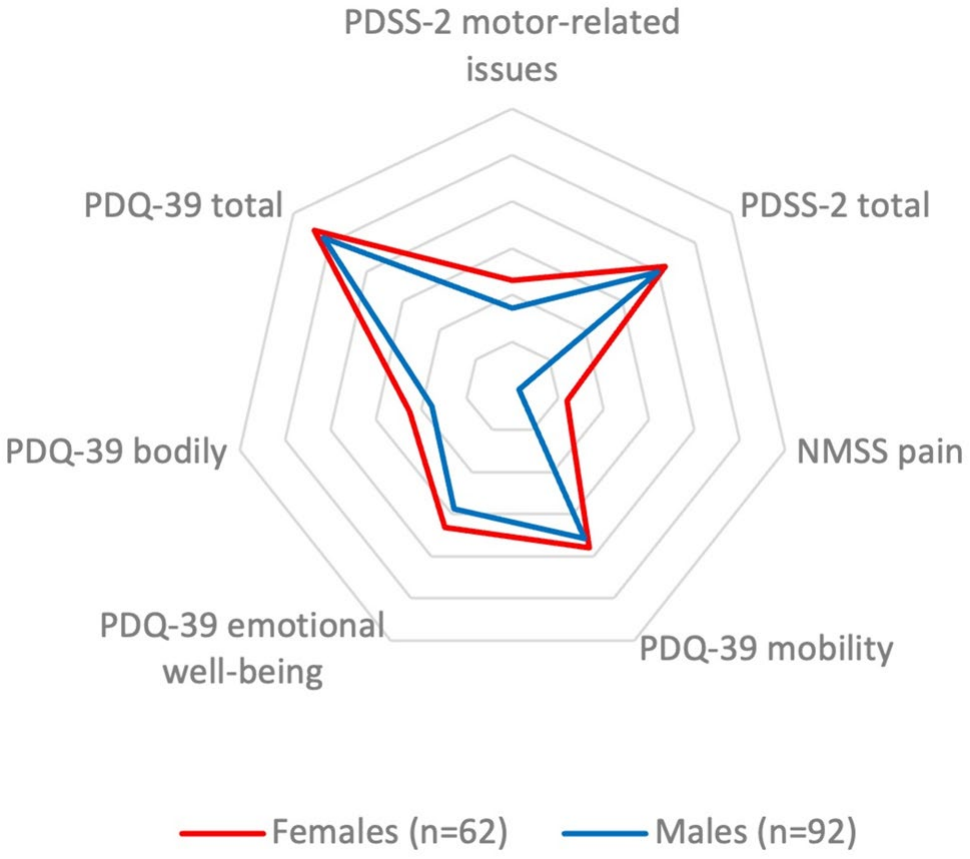
The figure shows the clinical differences in main clinical scales between the two sexes

Figure 1 shows the main clinical differences between the two sexes in the three clinical scales.

### *Comparison between poor sleepers (PDSS-2* < *18) and good sleepers (PDSS-2 ≥ 18) in each sex*

When comparing female good sleepers (PDSS-2 < 18, n = 31) with female poor sleepers (PDSS-2 ≥ 18, n = 29), the second had higher NMSS sleep [F = 8.06, p = 0.006], pain [F = 7.61, p = 0.008], total [F = 4.16, p = 0.047], and PDQ-39 mobility [F = 7.27, p = 0.009], ADL [F = 12.48, p = 0.001], emotional well-being [F = 6.83, p = 0.012], social [F = 4.68, p = 0.035], communication [F = 9.76, p = 0.003], bodily discomfort [F = 18.9, p < 0.001], and total scores [F = 10.53, p = 0.002] compared to good sleepers. When comparing male good sleepers (PDSS-2 < 18, n = 59) with male poor sleepers (PDSS-2 ≥ 18, n = 28), the second had higher H&Y [F = 9.98, p = 0.002], MDS-UPDRS III [F = 4.30, p = 0.042], NMSS sleep [F = 35.49, p < 0.001], mood/cognition [F = 8.55, p = 0.005], perception problems [F = 10.91, p < 0.001], attention/memory [F = 4.18, p = 0.044], gastrointestinal [F = 8.70, p = 0.004], urinary [F = 4.40, p = 0.039], pain [F = 8.80, p = 0.004], total [F = 20.05, p < 0.001], and PDQ-39 mobility [F = 19.88, p < 0.001], ADL [F = 9.01, p = 0.004], emotional well-being [F = 11.51, p = 0.001], cognition [F = 6.74, p = 0.011], communication [F = 5.35, p = 0.023], bodily discomfort [F = 13.13, p = 0.001], and total scores [F = 22.27, p < 0.001] compared to good sleepers.

### *Comparison between female poor sleepers (PDSS-2* < *18) and male poor sleepers (PDSS-2 ≥ 18)*

Female and male poor sleepers were similar in most clinical and demographic features. However, female poor sleepers had higher NMSS pain [F = 2.84, p = 0.047] and PDQ-39 bodily discomfort [F = 0.544, p = 0.024] scores compared to male poor sleepers.

### Correlation analysis in females with PD

Simple correlation analyses showed a positive correlation between PDSS-2 total scores and disease duration [R = 0.430, p = 0.001] and H&Y stage [R = 0.324, p = 0.002]. As for the NMSS scale, a strong correlation was found between PDSS-2 total scores and NMSS pain domain [R = 0.467, p < 0.001] and total scores [R = 0.461, p = 0.002]. Weaker positive correlations were found between PDSS-2 total scores and other subitems of the NMSS scale (cardiovascular [R = 0.260, p = 0.005] and urinary [R = 0.344, p = 0.011]). Finally, the PDSS-2 total scores positively correlated with the PDQ-39 total scores [R = 0.581, p < 0.001] and all the PDQ-39 subitems. No correlations were found between either PDSS-2 total or single subitems and LEDD values.

Table 2 shows in detail all the correlations found between each subitem of the PDSS-2 scale and other clinical scales in female PD patients.

**Table 2:** The table shows the correlations found between each subitem of the PDSS-2 scale and other clinical scales in female PD patients

	PDSS-2 motor	PDSS-2 quality of sleep	PDSS-2 dreaming distress	PDSS-2 fragmented sleep	PDSS-2 insomnia	PDSS-2 total scores
Disease duration	R = 0.268, *p = 0.046	NS	R = 0.390, *p = 0.003	R = 0.500, *p < 0.001	NS	R = 0.430, *p = 0.001
H&Y	NS	NS	NS	NS	NS	R = 0.324, p = 0.02
MDS-UPDRS III	NS	NS	NS	NS	NS	NS
LEDD	NS	NS	NS	NS	NS	NS
NMS cardiovascular	NS	NS	NS	NS	NS	R = 0.260, *p = 0.005
NMS sleep/fatigue	R = 0.312, *p = 0.020	NS	NS	R = 0.409, *p = 0.002	R = 0.340, *p = 0.012	NS
NMS mood/cognition	NS	R = 0.303, *p = 0.026	NS	NS	NS	NS
NMS perceptual	NS	NS	R = 0.387, *p = 0.004	NS	NS	NS
NMS attention/memory	NS	R = 0.282, p = 0.04	NS	NS	NS	NS
NMS gastrointestinal	NS	NS	NS	NS	NS	NS
NMS urinary	NS	R = 0.326, *p = 0.016	NS	R = 0.406, *p = 0.002	NS	R = 0.344, *p = 0.011
NMS sexual dysfunction	NS	NS	NS	NS	NS	NS
NMS pain	R = 0.428, *p = 0.002	NS	R = 0.316, *p = 0.020	NS	R = 0.304, *p = 0.025	R = 0.467, *p < 0.001
NMS smell	NS	NS	NS	NS	NS	NS
NMS weight	NS	NS	NS	NS	NS	NS
NMS sweating	NS	NS	NS	NS	NS	NS
NMS total	NS	R = 0.423, *p = 0.001	NS	R = 0.356, *p = 0.008	NS	R = 0.461, *p = 0.002
PDQ-39 mobility	R = 0.343, *p = 0.010	R = 0.315, *p = 0.020	NS	R = 0.29, *p = 0.033	R = 0.378, *p = 0.005	R = 0.472, *p < 0.001
PDQ-39 ADL	R = 0.351, *p = 0.009	R = 0.4443, *p = 0.001	NS	R = 0.334, *p = 0.013	R = 0.300, *p = 0.020	R = 0.509, *p < 0.001
PDQ-39 emotional well-being	R = 0.340, *p = 0.012	R = 0.312, *p = 0.018	R = 0.285, *p = 0.037	NS	R = 0.266, *p = 0.0266	R = 0.244, *p = 0.001
PDQ-39 stigma	NS	R = 0.259, *p = 0.05	R = 0.363, *p = 0.007	NS	R = 0.296, *p = 0.029	R = 0.333, *p = 0.014
PDQ-39 social support	R = 0.531, *p < 0.001	R = 0.309, *p = 0.023	NS	R = 0.342, *p = 0.011	NS	R = 0.550, *p < 0.001
PDQ-39 cognition	NS	R = 0.554, *p < 0.001	R = 0.339, *p = 0.012	R = 0.262, *p = 0.040	NS	R = 0.341, *p = 0.011
PDQ-39 communication	NS	NS	NS	NS	NS	R = 0.285, *p = 0.037
PDQ-39 bodily discomfort	R = 0.631, *p < 0.001	NS	R = 0.343, *p = 0.011	NS	R = 0.265, *p = 0.050	R = 0.568, *p < 0.001
PDQ-39 total	R = 0.384, *p = 0.004	R = 0.468, *p = 0 < 0.001	R = 0.383, *p = 0.004	R = 0.360, *p = 0.008	R = 0.339, *p = 0.008	R = 0.581, *p < 0.001

n, number; MDS-UPDRS, Movement Disorder Society Unified Parkinson’s Disease Rating Scale; PDSS-2, Parkinson’s Disease Sleep Scale-2; NMSS, Non Motor Symptoms Scale; PDQ-39; Parkinson’s disease Questionnaire. Age and disease duration are expressed in years. *Significant correlations using Pearson correlation analysis

**Supplemental Table 1 **shows the simple correlation analyses found between disease duration, LEDD values, and other non-sleep-related clinical scales in female PD patients.

### Correlation analysis in males with PD

As in females, simple correlation analyses showed a positive correlation between PDSS-2 total scores and disease duration [R = 0.283, p = 0.0101], and H&Y stage R = 0.283, p = 0.010]. Regarding the NMSS scale, a strong correlation was found between PDSS-2 total scores and NMSS sleep/fatigue domain [R = 0.625, p < 0.001] and total scores [R = 0.441, p < 0.001]. Weaker positive correlations were found between PDSS-2 scale and cardiovascular [R = 0.383, p < 0.001], mood/cognition [R = 0.280, p = 0.012], perception/visual hallucinations [R = 0.223, p = 0.047], gastrointestinal [R = 0.328, p = 0.003], urinary [R = 0.296, p = 0.008] domains. No correlation was found between PDSS-2 total scores and NMSS pain subitems. Finally, the PDSS-2 total scores positively correlated with the PDQ-39 total scores [R = 0.549, p < 0.001]. Differently than females, the PDSS-2 total scores positively correlated with only some of the PDQ-39 subitems (mobility [R = 0.478, p < 0.001], emotional well-being [R = 0.408, p < 0.001], cognition [R = 0.318, p < 0.004], and bodily discomfort [R = 0.555, p < 0.001]). Again, no correlations were found between either PDSS-2 total or single subitems and LEDD values.

Table 3 shows in detail all the correlations found between each subitem of the PDSS-2 scale and other clinical scales in male PD patients.

**Table 3:** The table shows the correlations found between each subitem of the PDSS-2 scale and other clinical scales in male PD patients

	PDSS-2 motor	PDSS-2 quality of sleep	PDSS-2 dreaming distress	PDSS-2 fragmented sleep	PDSS-2 insomnia	PDSS-2 total scores
Disease duration	NS	NS	NS	R = 0.365, *p = 0.001	NS	R = 0.283, *p = 0.010
H&Y	R = 0.248, *p = 0.027	NS	R = 0.240, *p = 0.032	NS	NS	R = 0.283 *p = 0.011
MDS-UPDRS III	NS	NS	R = 0.229, *p = 0.041	NS	NS	NS
LEDD	NS	NS	NS	NS	NS	NS
NMS cardiovascular	R = 0.404, *p < 0.001	R = 0.264, *p = 0.018	R = 0.252, *p = 0.024	NS	NS	R = 0.383, *p < 0.001
NMS sleep/fatigue	R = 0.505, *p < 0.001	R = 0.594, *p < 0.001	NS	R = 0.403, *p < 0.001	R = 0.262, *p = 0.019	R = 0.625, *p < 0.001
NMS mood/cognition	NS	NS	R = 0.279, *p = 0.012	NS	NS	R = 0.280, *p = 0.012
NMS perceptual	NS	NS	R = 0.244, *p = 0.029	NS	NS	R = 0.223, *p = 0.047
NMS attention/memory	NS	NS	R = 0.223, *p = 0.047	NS	NS	NS
NMS gastrointestinal	R = 0.339, *p = 0.002	R = 0.225, *p = 0.045	NS	NS	NS	R = 0.328, *p = 0.003
NMS urinary	R = 0.266, p = 0.017	NS	NS	NS	NS	R = 0.296, *p = 0.008
NMS sexual dysfunction	NS	NS	NS	NS	R = 0.245, *p = 0.029	NS
NMS pain	NS	NS	NS	NS	R = 0.245, *p = 0.028	NS
NMS smell	NS	NS	NS	NS	NS	NS
NMS weight	NS	NS	NS	NS	NS	NS
NMS sweating	NS	NS	NS	NS	NS	NS
NMS total	R = 0.330, *p = 0.003	R = 0.310, *p = 0.005	R = 0.268, *p = 0.016	R = 0.267, *p = 0.017	NS	R = 0.441, *p < 0.001
PDQ-39 mobility	R = 0.428, *p < 0.001	R = 0.262, *p = 0.019	R = 0.260, *p = 0.020	R = 0.299, *p = 0.007	NS	R = 0.478, *p < 0.001
PDQ-39 ADL	R = 0.299, *p = 0.007	NS	NS	R = 0.283, *p = 0.011	R = 0.222, *p = 0.048	R = 0.342, *p = 0.002
PDQ-39 emotional well-being	R = 0.296, *p = 0.098	R = 0.320, *p = 0.004	R = 0.251, *p = 0.025	NS	NS	R = 0.408, *p < 0.001
PDQ-39 stigma	NS	NS	R = 0.265, *p = 0.017	NS	NS	NS
PDQ-39 social support	NS	NS	NS	R = 0.342, *p = 0.011	NS	NS
PDQ-39 cognition	NS	R = 0.258 *p = 0.021	R = 0.311, *p = 0.005	R = 0.240, *p = 0.032	NS	R = 0.318, *p = 0.004
PDQ-39 communication	NS	NS	R = 0.266, *p = 0.017	NS	NS	NS
PDQ-39 bodily discomfort	R = 0.496, *p < 0.001	R = 0.416, *p < 0.001	R = 0.233, *p = 0.047	R = 0.228, *p = 0.042	R = 0.341, *p = 0.002	R = 0.555, *p < 0.001
PDQ-39 total	R = 0.402, *p < 0.001	R = 0.348 *p = 0.002	R = 0.336, *p = 0.002	R = 0.388, *p < 0.001	R = 0.235, *p = 0.036	R = 0.549, *p < 0.001

n, number; MDS-UPDRS, Movement Disorder Society Unified Parkinson’s Disease Rating Scale; PDSS-2, Parkinson’s Disease Sleep Scale-2; NMSS, Non Motor Symptoms Scale; PDQ-39; Parkinson’s disease Questionnaire. Age and disease duration are expressed in years. *Significant correlations using Pearson correlation analysis

**Supplemental Table 1** shows the correlation analyses found between disease duration, LEDD values, and other non-sleep-related clinical scales in male PD patients.

## Discussion

This study investigated extensively the impact of sleep on motor and non-motor symptoms and HR-QoL in a wide cohort of PD patients, finding that women experienced more severe sleep problems than their male counterparts. Furthermore, we found a different association between sleep, NMS, and HR-QoL in the two sexes, suggesting different etiopathogenesis and the need for tailored therapeutic approaches.

Sleep disturbances are one of the most common NMS of PD. Prior studies have found sex-dimorphic differences in PD patients’ sleep [16]. As in the general population, RLS is more common in female PD patients than in males, oppositely to RBD, which has a male predominance [16, 17]. Nevertheless, very few studies have investigated the influence of sex in each domain of the entire PD nocturnal sleep experience. A prior study that explored sleep disturbances in a large cohort of PD patients already found that females with PD had overall poor quality and efficiency of sleep compared to their male counterparts [18]. However, to our knowledge, this was the first study investigating sex-specific sleep patterns using the PDSS-2—a reliable tool encompassing the wide range of nighttime PD motor and non-motor symptoms. Furthermore, the PDSS-2 allows us to identify significant sleep problems based on a clinically defined cut-off score of 18 out of 60, obtained in the European PD population [13].

First, we observed that female PD patients exhibited higher average PDSS-2 scores and, using the clinically defined cut-off (18/60), a greater prevalence of individuals experiencing poor sleep quality than males. Notably, almost half of female PD patients could be labeled poor sleepers (48.3%), compared to about one-third of males (32.2%). Through an analysis of each domain of the PDSS-2 scale, we then discerned that the sleep disturbances most affecting females were those related to nocturnal motor problems, i.e., restlessness of the limbs, nocturnal akinesia, OFF-dystonia, painful cramps, and tremor on waking. Finally, we found that sleep disturbances were differentially correlated with NMS and HR-QoL in the two sexes.

Significative correlations were observed between sleep disturbances and NMS across the entire PD population, in accordance with previous findings [19], indicating that sleep problems could be a marker of frailty in both sexes. Indeed, the PDSS-2 significantly correlated with total values of the NMSS and specific dimensions, such as urinary disturbances in both sexes. However, some sex-specific differences emerged. Pain, a disabling and often underestimated PD NMS, correlated with global sleep experience and specific domains such as nocturnal motor-related problems, dreaming distress, and insomnia, mostly in females. Both pain and sleep disturbances arise from the common involvement of non-dopaminergic brainstem nuclei, such as the dorsal raphe nucleus and the reticular formation, mirroring a common trajectory of synucleinopathy [20]. Pain can alter sleep architecture, causing sleep fragmentation, decreased sleep efficiency, reduced REM sleep, and increased N1 sleep stage, whereas reduced sleep can lower the pain threshold [20]. As in the general population, pain greatly burdens PD female patients [21]. The reasons underlying the greater load of painful symptomatology in this population are various, encompassing differences in muscle mass, which could predispose to pain with musculoskeletal features; lower levels of gonadal sex hormones in the post-menopausal period with consequences on pain perception; differences in the notion of self, resulting in deeper attention to symptoms [22]. Furthermore, consistently with a peculiar vulnerability to levodopa-induced motor and non-motor complications, females with PD are reported to suffer especially with fluctuations-related pain, such as pain related to OFF-dystonia [23], one of the most disturbing PD sleep motor-related issues.

Unlike females, male sleep disturbances correlated with a range of NMS, such as cardiovascular and gastrointestinal issues, cognitive dysfunction, and perception disturbances rather than pain. Cardiovascular symptoms, together with urinary and gastrointestinal issues, may suggest the existence of autonomic dysfunction, which is closely related to sleep disturbances due to shared brainstem neuropathology [24]. Dysautonomic dysfunction, constipation, cognitive decline, and neuropsychiatric manifestations are markedly greater in PD patients with RBD, reflecting a more severe and widespread underlying neurodegenerative process [25]. RBD is more prevalent [16] or at least more clinically expressed in the male PD population [26]. Although speculative, since the lack of a polysomnographic confirmation, the different correlations found between NMS and sleep among the two sexes might suggest a different underlying predominant basis of the sleep disturbance itself, possibly involving RBD in males and a greater burden of fluctuations-related pain in females.

Subsequently, we uncovered how sleep disturbances profoundly impact the HR-QoL in both sexes, but especially in females with PD. Previous studies have highlighted a more substantial impact on daily activities and worse HR-QoL in females with PD compared to males, especially regarding bodily discomfort, stigma, and emotional well-being [27, 28]. When examining each aspect of the affected HR-QoL through the various domains of the PDQ-39 scale, it became evident that almost every domain correlated with nocturnal sleep disturbances across the entire PD population, spanning from global mobility and ADL to bodily discomfort, emotional well-being, and cognition. A bidirectional association links mood disorders and cognition to sleep disturbances in PD; indeed, whereas depression and anxiety are among the main determinants of sleep quality, sleep disturbances significantly impact attention and executive function [29]. However, we found specific aspects of the HR-QoL correlated to sleep only in females, namely stigma, social support, and communication.

Ultimately, in accordance with the sex-specific correlations found between NMS and sleep problems, on the one hand, we found that female poor sleepers had higher pain symptomatology compared to good sleepers. On the other hand, males showed worse motor functioning, cognition, attention/memory, perception disturbances, and gastrointestinal disturbances compared to good sleepers. Finally, in both sexes, poor sleepers showed a worse HRQoL than the good ones, regardless of age and disease duration, despite higher rates of bodily discomfort scores in female poor sleepers compared to males.

The reasons underlying the heavier burden of sleep disturbances in females with PD remain to be fully elucidated. A large body of evidence illustrates the beneficial role of female sex hormones, particularly estradiol, on the degenerating nigral dopaminergic system [4, 30]. However, many NMS, including pain, mood disorders, and sleep disturbances, arise from other neurotransmitter systems and neuropathological changes [31] and might be protected to a lesser extent by female sex hormones. It is worth noticing that none of the main sleep disturbances were affected by dopaminergic treatment, neither in females nor in males, further supporting the relevance of the involvement of different neurotransmitter systems. Additionally, it is important to consider that other factors such as genetic predisposition, environmental factors, social and cultural influences, differences in healthcare-seeking behavior, and access to treatment account for a huge amount of sex-specific differences found in PD [32, 33]. Women with PD experience a greater challenge with access to healthcare delivery and receive lesser social support compared to males [34], with possibly further implications on various facets of their unique symptomatology, including nocturnal sleep.

Further studies are needed to better understand the relatively lower burden of sleep problems in males with PD. Indeed, we found that only 32.2% of males with PD could be considered as poor sleepers according to the cut-off for the European PD population proposed. Whether this data might result unexpectedly, considering that the literature shows that the vast majority of patients with PD suffer from some form of sleep dysfunction [3], data on the severity of such disturbances using validated cut-off and clinical scales is scarce. Using a cut-off of 15 on the PDSS-2, previous Authors have found rates of poor sleepers ranging from 45.9 to 47.8% among PD patients [35, 36]. Considering both females and males with PD, we found an overall rate of 38.8%, close to those existing, considering the higher cut-off used on the PDSS-2 scale (18) [13].

Limitations of this study include the relatively small sample size, the cross-sectional design, which did not allow an evaluation of the longitudinal progression of sleep disturbances over time, and, most importantly, the lack of a polysomnographic examination of sleep disturbances.

Despite these limitations, this study provides compelling evidence that females with PD contend with more severe sleep disturbances and diminished HR-QoL compared to their male counterparts. These findings indicate sleep disorders as a marker of frailty in female PD patients, independent of age, disease duration, and dopaminergic treatment.

Further research is needed to better understand the impact of sleep disturbances in female patients with PD and help provide valuable insights for personalized treatment approaches for this underserved population.

## Supplementary Information

Below is the link to the electronic supplementary material.

Supplementary file1 (DOCX 155 kb)

## Data Availability

The datasets generated during analysis are available from the corresponding author upon reasonable request.
