# Supplementary material for: Sex differences in Parkinson’s disease-related non motor symptoms: a focus on sleep problems
Source: Acta Neurol Belg. 2024 Apr 4;124(5):1525–34. doi: 10.1007/s13760-024-02535-8 (PMC11614980; doi:10.1007/s13760-024-02535-8)
Supplement: Supplementary file 1 — Supplementary file1 (DOCX 155 kb) [file 13760_2024_2535_MOESM1_ESM.docx]

**Supplemental Table 1.** The table shows the correlations found between disease duration, LEDD values, and non-sleep-related clinical scales in female (F) and male (M) patients with PD patients.

|  | Sex | Disease Duration | LEDD | H&Y | MDS-UPDRS III | NMSS total | PDQ-39 |
| --- | --- | --- | --- | --- | --- | --- | --- |
| Disease Duration | F | 1 | R=0.687, *p<0.001 | R=0.680, *p<0.001 | R=0.660, *p<0.001 | R=0.311, *p=0.018 | R=0.451, *p<0.001 |
|  | M | 1 | R=0.642, *p<0.001 | R=0.641, *p<0.001 | R=602, *p<0.001 | NS | R=0.393, *p<0.001 |
| LEDD | F | R=0.687, *p<0.001 | 1 | R=0.631, *p<0.001 | R=0.600, *p<0.001 | R=0.328, *p=0.003 | R=0.687, *p<0.001 |
|  | M | R=0.642, *p<0.001 | 1 | R=0.861, *p<0.001 | R=0.499, *p<0.001 | NS | R=0.227, *p=0.039 |
| H&Y | F | R=0.796, *p<0.001 | R=0.600, *p<0.001 | 1 | R=0.796, *p<0.001 | R=0.600, *p<0.001 | R=0.631, *p<0.001 |
|  | M | R=0.641, *p<0.001 | R=0.632, *p<0.001 | 1 | R=0.861, *p<0.001 | R=0.527, *p<0.001 | R=0.641, *p<0.001 |
| MDS-UPDRS III | F | R=0.660, *p<0.001 | R=0.600, *p<0.001 | R=0.796, *p<0.001 | 1 | R=0.530, *p<0.001 | R=0.514, *p<0.001 |
|  | M | R=0.602, *p<0.001 | R=0.499, *p<0.001 | R=0.861, *p<0.001 | 1 | R=0.428, *p<0.001 | R=0.522, *p<0.001 |
| NMSS total | F | R=0.311, *p=0.018 | R=0.382,  *p=0.003 | R=0.600,  *p<0.001 | R=0.530, *p<0.001 | 1 | R=0.786,  *p<0.001 |
|  | M | NS | NS | R=0.342, *p=0.001 | R=0.428 *p<0.001 | 1 | R=0.617, *p<0.001 |
| PDQ-39 | F | R=0.451, *p<0.001 | R=0.473, *p<0.001 | R=0.628, *p<0.001 | R=0.514, *p<0.001 | R=0.786, *p<0.001 | 1 |
|  | M | R=0.393, *p<0.001 | R=0.227, *p=0.039 | R=0.527, *p<0.001 | R=0.522, *p<0.001 | R=0.617, *p<0.001 | 1 |

LEDD, Levodopa Equivalent Daily Dose; MDS-UPDRS, Movement Disorder Society Unified Parkinson’s Disease Rating Scale; NMSS, Non Motor Symptoms Scale; PDQ-39; Parkinson’s disease Questionnaire. Disease duration are expressed in years. *Significant correlations using Pearson correlation analysis.
